# Cost-Effectiveness of Screening and Treating Chronic Hepatitis C Virus Infection in Zimbabwe

**DOI:** 10.3390/ijerph22040509

**Published:** 2025-03-27

**Authors:** Blessing Dzingirai, Leolin Katsidzira, Maarten J. Postma, Marinus van Hulst, Nyashadzaishe Mafirakureva

**Affiliations:** 1 Department of Health Sciences, University Medical Center Groningen, University of Groningen, 9700 RB Groningen, The Netherlands; m.j.postma@umcg.nl (M.J.P.); m.van.hulst@rug.nl (M.v.H.); 2 Department of Pharmacy and Pharmaceutical Sciences, University of Zimbabwe, Harare P.O. Box MP 167, Zimbabwe; 3 Department of Medicine, College of Health Sciences, University of Zimbabwe, Harare P.O. Box MP 167, Zimbabwe; katsidzira.leo@gitclinic.co.zw; 4 Department of Clinical Pharmacy and Toxicology, Martini Hospital, 9728 NL Groningen, The Netherlands; 5 Sheffield Centre for Health and Related Research, School of Medicine and Population Health, University of Sheffield, Sheffield S10 2TN, UK; mafirakurevan@gmail.com

**Keywords:** hepatitis C virus, cost effectiveness analysis, low- to middle-income countries, directly acting antivirals, Zimbabwe

## Abstract

Background: The aim of this study was to assess the cost effectiveness of a screening and treatment intervention approach for chronic HCV infection in Zimbabwe. Methods: Using a decision tree and a validated Markov model, we estimated the lifetime costs and health effects of screening for and treating HCV infections from a healthcare perspective. We evaluated three screening strategies, namely the following: i. no screening; ii. screening among the general population; and iii. screening among high-risk groups. Incremental cost effectiveness ratios were calculated for the strategies that were not dominated. We used deterministic and probabilistic sensitivity analyses to explore the impacts of parameter uncertainty on cost effectiveness outcomes. Results: The strategy of screening among high-risk groups and treating with sofosbuvir/velpatasvir had an incremental cost of USD 1201 and incremental quality-adjusted life years (QALY) of 2.01, yielding an incremental cost effectiveness ratio (ICER) of USD 604 per QALY gained as compared to no screening. The ICER was below the 0.5 times the gross domestic product per capita parameter (USD 796), making the intervention potentially cost effective. The strategy to screen among the general population was dominated, because it costed more and resulted in fewer QALYs than its comparators. Conclusions: Screening for HCV among high-risk populations followed by treatment using sofosbuvir/velpatasvir is cost effective under the assumptions made in this study.

## 1. Introduction

Hepatitis C virus (HCV) infection remains a public health challenge, with 58 million people living with the disease globally [1]. One-sixth (8 million) of the number of chronically infected live in Africa [1]. Chronic HCV infection can cause liver cirrhosis, hepatocellular carcinoma, and death. In 2022, it was estimated that sixty-seven thousand people lived with chronic HCV [2], and the disease resulted in 582 deaths in Zimbabwe [3]. The World Health Organization (WHO) Global Health Sector Strategy on Viral Hepatitis has set targets to achieve the elimination of HCV by 2030 [4]. The elimination plan includes global targets to have 90% of the people infected diagnosed and 80% treated. Very few countries are on track to meet these set targets by 2030 [5]. It is estimated that the number of people treated needs to be increased by almost tenfold, from the current 750,000 per year to 7.2 million per year [5]. Zimbabwe, like many low- to middle-income countries (LMICs), lags behind on the HCV diagnosis and treatment targets [6,7,8]. It has been reported that in Zimbabwe, 16% and 0.69% [9] of the infected were diagnosed and are on HCV treatment, respectively.

One of the major developments in the treatment of HCV was the discovery of directly acting antivirals (DAAs), which are highly effective and safe [10,11]. Although the prices of DAAs have significantly decreased, the cost of treatment remains a major barrier to access in low-income countries such as Zimbabwe. A study in Zimbabwe reported a cost of USD 1400 per 12-week course of generic sofosbuvir/velpatasvir [12]. This represents a 96% decrease from the cost of the originator brands [13], but is still prohibitive to the Zimbabwean population, which has an average household monthly income of USD 370 [14] and very low health insurance coverage [15]. Other barriers to access to HCV treatment are a lack of patient and provider knowledge on HCV, limited access to diagnostics, and complex care pathways [16]. Additionally, some groups that are at risk of HCV infection, such as people who inject drugs (PWID), people living with HIV, and people who are incarcerated, may fail to access screening and treatment services due to stigmatization, criminalization, and their mistrust of the healthcare system [17,18,19].

In 2019, the Ministry of Health in Zimbabwe developed a strategic plan for the elimination of HCV [20]. As part of the strategies, sofosbuvir/velpatasvir (sof/vel) and sofosbuvir/ledispasvir were included in the national treatment guidelines and essential medicines list [21]. To fully implement the strategic plan, there is need to screen and treat many people. The health impacts, costs, and cost effectiveness of such an intervention are not known in this setting. Investing in the screening and treatment of HCV will result in displacement of health benefits in the healthcare system, represented as the opportunity cost [22]. Cost effectiveness analysis provides a systematic method to determine if investing in the “new intervention” is good value for money as compared to the opportunity cost [22]. Additionally, cost effectiveness evidence is required by policy makers to aid strategic purchasing of diagnostics, DAAs, and other health services needed in HCV care [23]. Cost effectiveness analysis results can be used to determine which group of people need the services most, what are the most suitable prices, and what is the most efficient service delivery [24]. Strategic purchasing of health services ensures efficiency, quality, and equity in allocation of resources [23]. In our study, we evaluated the cost effectiveness of screening and treating chronic HCV infections using sof/vel in Zimbabwe. The counterfactual was assumed to be no screening, because routine HCV screening is not available in the public sector [20]. This information is timely and useful for policymakers as they implement the strategic plan to eliminate HCV in Zimbabwe.

## 2. Materials and Methods

### 2.1. Screening and Treatment Strategies

The intervention included screening for HCV and treatment using DAAs following a previously published model of care [12]. The screening included serological testing using rapid diagnostic tests (RDTs), which detect the presence of HCV antibodies. Individuals returning positive results on the serological tests underwent a nucleic acid test (NAT) to confirm chronic HCV infection. Individuals with confirmed chronic HCV infection underwent pretreatment assessment, including fibrosis assessment and other pretreatment laboratory tests. Eligible individuals were initiated on treatment using sofosbuvir 400 mg/velpatasvir 100 mg (sof/velp). Treatment response was assessed 12 weeks after completion of treatment, using quantitative HCV NAT. A cure is defined as a sustained virologic response (SVR), which is when HCV ribonucleic acid is not detectable in plasma 12 weeks after treatment completion.

We evaluated the cost-effectiveness of the following:

1. no screening, no treatment (comparator);
2. screening the general population and treating with sof/vel;
3. screening the high-risk populations and treating with sof/vel.

The general population referred to all the individuals 18 years and older. The high-risk populations were defined as people aged 50–79, commercial sex workers (CSWs), people who inject drugs (PWID), and men who have sex with men (MSM), as informed by literature [6,25]. The no screening option reflects the status quo where individuals are not routinely tested for HCV in Zimbabwe [6].

### 2.2. The Model

#### 2.2.1. Screening

The decision tree used to evaluate the screening strategies is shown in Figure S1 in the Supplementary Materials. The decision tree was populated using the estimates of the size of the general population and high-risk cohorts, HCV prevalence (serology and viremia) for both cohorts, and costs of RDT and NAT HCV tests. The size of the general population and number of people aged 50–79 were obtained from the Zimbabwe 2022 census report [26]. The number of CSWs was obtained from literature [27]. The numbers for PWID and MSM were assumed to be one thousand each, based on the Zambian estimates of nine hundred [28]. The estimates for the prevalence of HCV and costs of RDTs and HCV NATs were obtained from literature [12,29]. The outcomes from the screening were the number of people with chronic infection and cost of case finding.

#### 2.2.2. Markov Model

**Figure 1:**
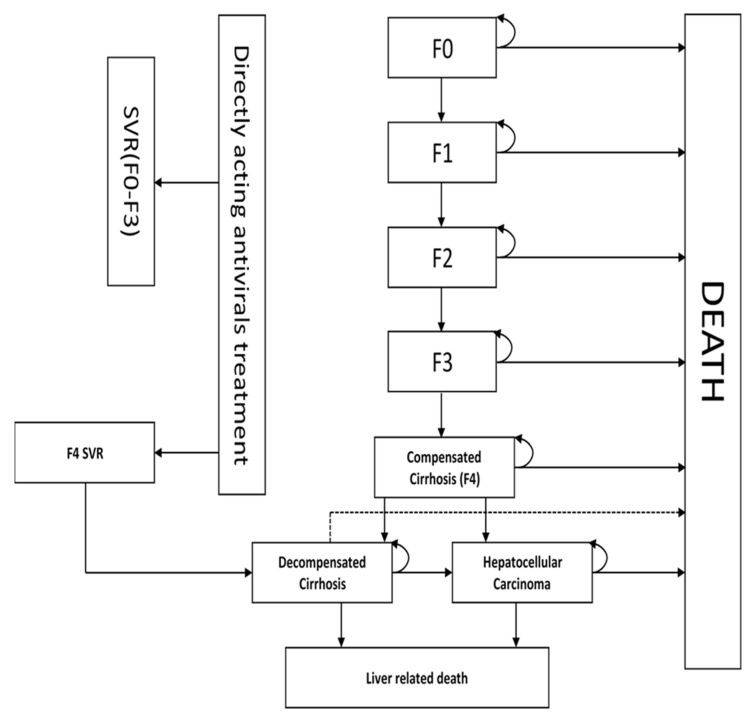
Markov model for hepatitis C virus chronic infection. F0–F4 METAVIR scores, F0 (no fibrosis), F1 (portal fibrosis without septa), F2 (portal fibrosis with few septa), F3 (numerous septa without fibrosis), and F4 (compensated cirrhosis). SVR—sustained virologic response.

To evaluate the cost effectiveness of screening and treatment of HCV infections, we used a previously used and validated Markov model [30]. The model (Figure 1) depicts the progression of chronic HCV infection using the metavir classification. According to the natural history of chronic HCV infections, an individual progresses from F0 (no fibrosis), to F1 (portal fibrosis without septa), to F2 (portal fibrosis with few septa), to F3 (numerous septa without fibrosis), and to F4 (compensated cirrhosis). Individuals in the F4 stage can progress to advanced health states of liver disease, which are decompensated cirrhosis (DC) and hepatocellular cancer (HCC) and eventually liver-related death. The age-specific mortality rate was estimated using life tables for Zimbabwe [31]. We assumed and used a one-year cycle, which is reflective of the slow progression of the disease. The natural history of HCV predicts the slow progression of fibrosis to cirrhosis taking 30–40 years [32], and most HCV cost effectiveness models have used the 1-year cycle [33,34,35]. In the model, patients are treated with sof/velp during the first cycle. The untreated patients and those that failed to attain SVR after treatment were assumed to progress to advanced health states, according to natural history of the disease. We also assumed that individuals attaining SVR from the F4 state could progress to the advanced states of the disease. Liver transplantation was not included in the model because it is currently not available in Zimbabwe.

### 2.3. Model Parameters

The inputs required for the Markov model included patient characteristics, DAA SVR rates, utility scores, transition probabilities, and costs. The estimates of the parameters used in the base case analysis are shown in Table S1 in the Supplementary Materials.

#### 2.3.1. Patient Characteristics

The initial distribution of patients to different health states was obtained from expert opinion. The information obtained from the experts is shown in the Supplementary Materials, Table S1. Another important characteristic of the cohort was the starting age at entry into the model. We obtained the start age for base case analysis [61 (95% CI, 54.5–67.8) years] by analyzing data from the clinical registry of HCV patients at a private hospital in Zimbabwe. We assumed the high-risk population to have a lower start age of 55, due the contribution of a lower age of the CSW group (15–49) [27].

#### 2.3.2. DAAs SVR Rates

The SVR rate for sof/velp (94.2% (95% CI 90.7–97.7) was obtained from literature [36]. The SVR rate for sof/velp was adopted from a systematic review and meta-analysis that pooled data for five clinical trials and 1277 patients.

#### 2.3.3. Transition Probabilities

The annual transition probabilities for F0–F4 transitions for the general population cohort were obtained from a meta-analysis [37] (see Table S1 in the Supplementary Materials). The study reviewed 111 studies with a total of 42,693 patients. Stage-constant and stage-specific fibrosis progression rates were estimated and reported for each study. We converted the reported fibrosis progression rates to annual transition probabilities using published guidance [38]. The transition probabilities for the high-risk cohort were assumed to be slightly higher than those of the general population, due to higher risk of HIV co-infection in these populations. HIV co-infection may accelerate progression of fibrosis [39,40]. We obtained transition probabilities for the high-risk cohort from a meta-analysis that estimated the transition probabilities of 3567 HIV/HCV co-infected patients from 17 studies [40]. The annual transition probabilities from F4 to the advanced states of liver disease (DC and HCC) were not included in the meta-analyses and were obtained from other literature sources [33].

#### 2.3.4. Utilities

The utilities required to estimate the quality-adjusted life years (QALYs) for each health state in the model were obtained from a clinical trial conducted in Central and West Africa [41]. In the clinical trial, HCV patients were treated with sofosbuvir/ribavirin or sof/led. Health-related quality of life scores were assessed before treatment initiation (at enrollment), during treatment at weeks 2, 4, 8, and 12, and after treatment (weeks 24 and 36). In this evaluation, the health-related quality of life scores before and after treatment from the clinical trial were used to derive the utility weights that we used to obtain the QALYs.

#### 2.3.5. Costs

We included the drug and healthcare costs for each health state in the Markov model. We assumed that the costs relevant in the model were the costs specifically related to HCV, and so we did not include costs for the management of other comorbidities. We also assumed that individuals in the F0–F3 states in the no-treat option incurred no HCV-related costs due to its asymptomatic nature in the early stages. HCV-related costs were incurred in the F4 (compensated cirrhosis), DC, and HCC states. To determine the costs for the F4, DC, and HCC health states, we used micro-costing. The details of the management are recorded in the Supplementary Materials, Section S4, Table S2. The drug and healthcare costs for DAA treatment were obtained from a study that was conducted in Zimbabwe [12]. In that study, the costs for HCV screening and treatment were estimated at a tertiary hospital using activity-based costing from a healthcare sector perspective.

### 2.4. Cost Effectiveness Analysis

We estimated the total discounted costs and health benefits for all the strategies from a healthcare perspective over a lifetime horizon. The health outcomes and the costs were all discounted at an annual rate of 3%. The QALYs were obtained by multiplying the utilities associated with a health state by the number of years spent in that health state. The results of the cost effectiveness analysis were reported as incremental cost effectiveness ratios (ICERs). Screening and treatment strategies were ranked by increasing discounted costs. ICERs were calculated for non-dominated strategies for comparison to the next best strategy. The ICER was obtained by dividing the incremental costs by incremental QALYs. The ICERs were compared to Zimbabwe’s 2023 per capita gross domestic product (GDP) of USD 1592 [42]. We also compared the ICERs to 0.5× GDP per capita, which is close to the opportunity cost-based cost effectiveness threshold (CET) for Zimbabwe, based on guidance from literature [43].

### 2.5. Sensitivity Analysis

In the scenario analyses, we considered the price of a 12 week supply of sof/velp of USD 270 [44], offered under the United Nations Development Program (UNDP) negotiated price and USD 550 [44] reported for the pooled procurement. We also evaluated the impact of changing the treatment regimen from sof/velp to sofosbuvir/ledipasvir on the ICERs. Sofosbuvir/ledipasvir is included in the Zimbabwean treatment guidelines as an alternative to sof/velp.

We performed deterministic sensitivity analysis (DSA) and probabilistic sensitivity analysis (PSA) to explore the impact of uncertainty in the model parameters on the ICERs for all the options. For the DSA, we varied the parameters across their 95% confidence intervals and presented the corresponding ICERs on a tornado diagram. If the 95% confidence interval of the parameter estimate was not available, we varied the parameter value by ±20%. We used 1000 Monte Carlo simulations to generate the PSA samples. The model parameters’ values were sampled from their predefined distributions. Per standard practice, the random values for transition probabilities and utilities were drawn from the beta distribution and costs from the gamma distribution. The random parameter values were used to compute the total costs and QALYs for all the scenarios. Also, considering the uncertainty in the CET, we estimated the probability of different options being cost effective over a range of CETs. The results were expressed as a cost effectiveness acceptability curve (CEAC).

## 3. Results

### 3.1. Screening

The decision tree predicted a total of 91,874 individuals living with chronic HCV from 8.6 million people screened in the general population and 53,317 individuals living with chronic HCV from 1.7 million people screened in the high-risk populations. The cost for case finding was USD 137 per person for the high-risk populations and USD 275 per person for general population screening.

### 3.2. Public Health Impacts of the Intervention

The model predicted a total of 54,878 patients experiencing cirrhosis and 8261 developing HCC and 9911 liver-related deaths in the no screening and no treatment strategy. The screening among the high-risk strategy resulted in 53,317 patients treated, yielding a reduction in cirrhosis cases to 488 and liver-related deaths to 20, at a total cost of USD 87.5 million. The strategy to screen for HCV in the general population resulted in 91,874 patients treated, yielding reduction in cirrhosis cases to 1095 and liver-related deaths to 44 at a total cost of USD 150 million. Table 1 shows the impact of the screening and treatment strategies on the number of people experiencing cirrhosis, DC-, HCC-, and HCV-linked deaths.

### 3.3. Base Case Analysis

The screening among high-risk populations and treatment with the sof/velp strategy resulted in an ICER of USD 604 per QALY gained when compared to the no screening strategy. The ICER was less than the 0.5 times GDP per capita parameter (USD 796) for Zimbabwe. The general population screening and treatment with the sof/velp strategy was dominated because it costed more and resulted in fewer health benefits than the screening among high-risk populations strategy. In the scenario analysis, changing the price of sof/velp to USD 550 and USD 270 reduced the ICER to USD 173 and USD 33 per QALY gained, respectively. Changing the DAA regimen to sof/led resulted in an incremental cost of USD 939 and QALYs of 1.95 per patient, yielding a reduced ICER of USD 481 per QALY gained for the screening strategy for high-risk patients. The total costs, QALYs, and ICERs per patient for the evaluated strategies and scenarios are shown in Table 2.

### 3.4. Sensitivity Analysis

**Figure 2:**
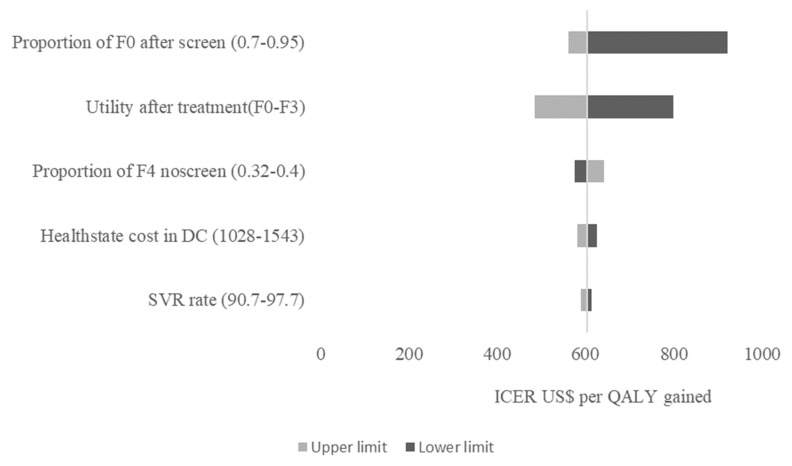
Tornado plot showing the one-way sensitivity for screening high-risk populations and treatment with sofosbuvir/velpatasvir vs. no screening and no treatment option. F0—metavir fibrosis health state F0, F3—metavir fibrosis health state F3, F4—metavir fibrosis health state F4, DC—decompensated cirrhosis, SVR—sustained virologic rate.

**Figure 3:**
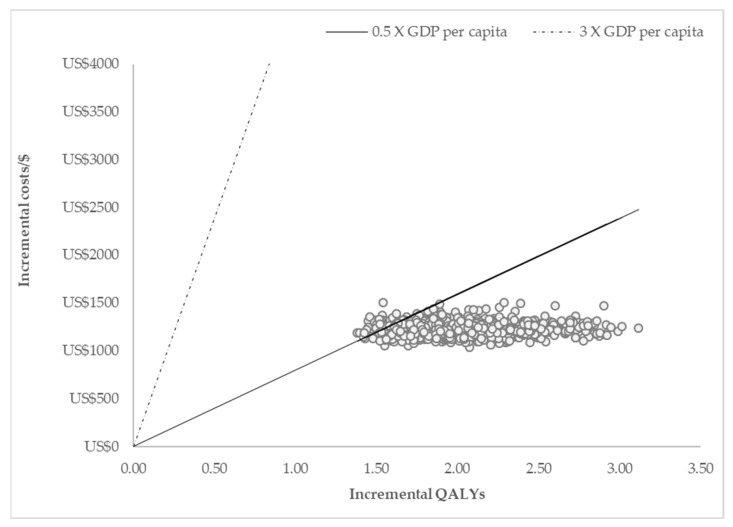
ICER scatter plot for the screening high-risk population and treatment with sofosbuvir/velpatasvir vs. no screening option. QALYs—quality-adjusted years, GDP—gross domestic product, USD—United States dollars.

**Figure 4:**
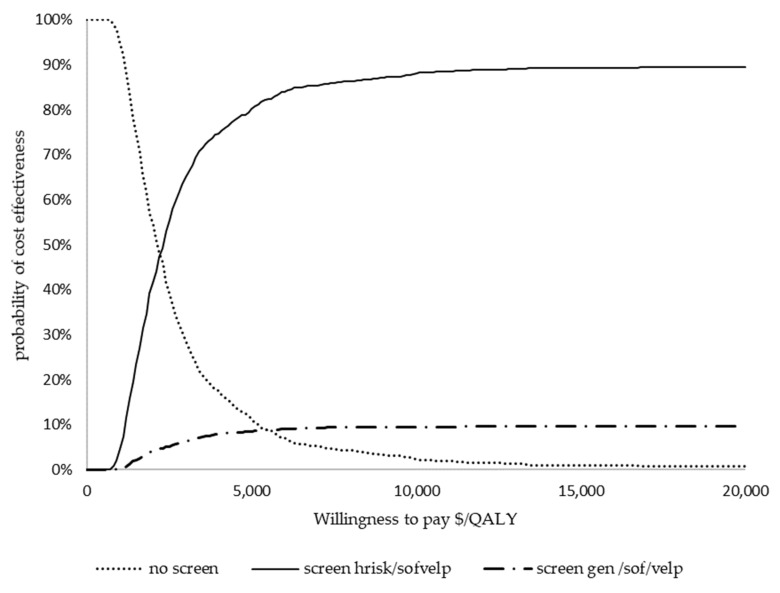
Cost effectiveness acceptability curves. QALY—quality-adjusted years, sof/velp—sofosbuvir/velpatasvir.

The impact of parameter changes on the ICER is shown in Figure 2. The parameters that had the greatest impact on the ICER were the proportion of patients in the F0 health state in the screened cohort, utility of F0–F3 after treatment, and proportion of patients in the F4 health state in the no screening cohort. The ICER scatter plot in Figure 3 shows the distribution of incremental costs and QALYs from 1000 Monte Carlo simulations of the comparison between screening high-risk populations, treating with sof/velp vs. no screening option. All the simulations fell below the GDP per capita, and 95% of the simulations were below the 0.5 times GDP per capita parameter. The probability of the strategies being cost effective over a range of CETs is shown in Figure 4. The CEAC shows that the strategy to screen in the high-risk population had a 100% probability of being cost effective at a willingness to pay of USD 520 per QALY gained, and screening in the general population strategy was dominated.

## 4. Discussion

In our study, we showed that screening and treating people in high-risk groups will reduce the occurrence of advanced disease, deaths and was cost effective. This is a favorable result for Zimbabwe, where liver cancer is on the rise and contributes 5.1% of all deaths due to cancer [45]. Although many etiologies are linked to liver cancer, the contribution of HCV is significant in Zimbabwe [46]. The ICER for the base case analysis estimated in this study of USD 604 per QALY gained was comparable to a similar study for Cameroon, Cotê de’Ivoire, and Senegal [33] and much higher than reported in other studies in LIMICs [47]. The possible reasons for higher ICERs can be high costs for DAAs in our study and a higher starting age of 61. When the start age was reduced to 40 years, the intervention became highly cost effective with ICERs (USD 308), well below the GDP per capita (USD1592) for Zimbabwe. The reduction in the ICERs was driven by increases in the incremental QALYs. This is evidence that if screening and treatment of HCV infection is carried out early, it increases the health benefits.

Although many studies have provided evidence for the value for money for screening and treating HCV using DAAs in LMICs, the question that remains is if the governments can afford these interventions. In this study, we estimated that the total cost to screen and treat HCV among the high-risk population in Zimbabwe was USD 87.5 million. This amount is relatively high and represents 14.2% of the 2024 health budget in Zimbabwe. To achieve the WHO targets by 2030, there is need for investment in HCV screening and treatment of more than USD 14 million per year over the next six years. The need for significant investment to achieve HCV elimination has been reported in other countries [48,49,50]. However, the significant investment is justified by the substantial health and economic benefits that can be realized from the interventions [48,49,50].

Other ways of reducing the costs of HCV care include adoption of a shorter 8-week course of treatment, use of HCV point-of-care assays, and integration of HCV care into existing health programs. Several studies have reported the non-inferiority of an 8-week course of sofosbuvir/ledispasvir in genotype 1-infected, non-cirrhotic patients with less than 6 million IU/mL viral load [51,52,53]. A systematic review and meta-analysis of the efficacy of the 8-week sofosbuvir/daclatasvir regimen in LMICs reported a pooled SVR12 of 91–97%, which was similar to ones used in this analysis [54]. Glecaprevir/pibrentasvir also has proven efficacy and is recommended for an 8-week course in all HCV genotypes [55]. Cost-savings accrued from the shortened DAA course has the potential to reduce DAA costs and make the intervention more cost effective. In this study, we showed that reducing the cost of DAA to USD 550 will reduce the ICER to USD 173 per QALY gained. However, further research on the real-world effectiveness of shortened DAA regimens in different clinical settings like HIV co-infection is required [54]. Also, there is need to explore cost effectiveness of the shortened DAA regimens, taking into account the high cost of glecaprevir/pibrentasvir. HCV point-of-care assays for diagnosis and pre-treatment assessments are less expensive and have shorter turnaround times than laboratory-based assays [56]. This reduces costs and simplifies treatment algorithms. Integration of HCV care into existing health programs such HIV/AIDS care enables sharing of existing infrastructure, which reduces costs [57]. In Zimbabwe, there is a successful HIV/AIDS program. HCV screening and treatment services can be offered from the same facilities with HIV/AIDS services, reducing costs.

In this study, we report that screening and treatment of chronic HCV among high-risk populations is cost effective. The high-risk populations include CSW, PWID, and MSM. However, there is need to address some barriers to access HCV care in these populations, because these populations are marginalized, stigmatized, and criminalized in Zimbabwe [57]. Lessons can be drawn from HIV/AIDS care, where innovative ways have been employed successfully to link the key populations to health services. The innovative ways include community-based mobilization and linkage to care, low-threshold access such as less registration details, peer-driven treatment programs, and flexible appointment times [58,59].

The major strength of this study is that we estimated the unit costs of screening and treatment of HCV from a micro costing study in Zimbabwe. Micro costing provides detailed cost information, detailing resource use in the intervention. The other strength of the study is that it is the first and timely cost effectiveness study on the screening and treatment of HCV in Zimbabwe using DAAs. The study is timely because it can be used to inform the implementation of the national viral hepatitis elimination strategy.

Our study had limitations. Firstly, the initial proportions of patients in different health states and the costs of managing cirrhosis and HCC were obtained from expert opinion. However, the experts are very experienced in the management of these patients in Zimbabwe, hence the estimates are a good representation of what is available. Also, we adopted health-related quality-of-life scores from a clinical trial that was carried out in another country. The health-related quality-of-life scores vary between different groups of people, depending on how they value health. However, the lack of country-specific data to inform cost effectiveness analyses is common in many LMICs [60]. Lastly, we used a GPD per capita-based CET. The use of GDP per capita as the CET has been criticized because of lack of scientific evidence to support its derivation [61,62]. It has been suggested that one to three times the GDP threshold is too high for LMICs where resources are constrained. Recommendations have been made for country-specific thresholds that represent opportunity costs of funding health interventions. One study estimated the supply-side cost per disability-adjusted life year (DALY) averted in South Africa as USD 3015 [63]. However, we cannot adopt the one from South Africa, due to different health spending and income levels. Other options to address the limitations of the GDP-based thresholds include budget impact analysis with presentation of ICERs and use of a league of table of health interventions [44]. The latter has been used to estimate the cost per DALY for a number of health interventions in Zimbabwe [64]. Although the study is very old, the method can be updated and used to determine context-specific CETs for use by decision makers.

Despite the mentioned limitations, this study provides valuable information to the Ministry of Health in Zimbabwe. The Ministry intends to screen and treat HCV as part of its strategic plan to eliminate viral hepatitis. Estimates of costs can be used to evaluate budget impacts of such interventions. The positive cost effectiveness result supports implementation of public health-based HCV screening and treatment programs. The results from this study can also be used in the development of an investment case for sourcing funds from developmental partners to scale up HCV screening and treatment. In addition to its direct value to Zimbabwe, the study also provides valuable information to other LMICs.

## 5. Conclusions

This study demonstrated that, compared to no screening and no treatment, screening among high-risk populations and treatment of HCV using sof/velp reduce morbidity and mortality due to HCV and is potentially cost effective in Zimbabwe. However, the intervention may have a significant impact on the healthcare budget, which needs to be taken into consideration by decision makers.

## Figures and Tables

**Table 1:** The estimated public health impacts of different options of screening and treatment of chronic HCV.

	No Screening/No Treatment Option	Screening High-Risk Population and Treatment with Sof/Velp Option	Screening General Population and Treatment with Sof/Velp Option
Total number screened	-	1,708,892	8,602,410
Total number treated	-	53,317	91,874
Total number cirrhosis	35,831	452	1013
Total number decompensated cirrhosis	19,047	36	82
Total number HCC	8261	12	35
Number of liver related deaths	9911	20	44

**Table 2:** The estimated discounted lifetime costs and QALYs and ICERs for different screening and treatment strategies.

				Incremental		ICER
	Strategy	Costs/USD	QALYs	Costs/USD	QALYs	USD/QALY
	**Base Case**					
	No screening, no treatment	565	3.41	-	-	-
	Screen high-risk populations and treat with sof/velp	1778	5.42	1213	2.01	604
	Screen general population and treat with sof/velp	1910	4.38	132	−1.04	Dominated
	**Scenario 1 sof/velpprice**					
**USD 270**	No screening, no treatment	565	3.41	-	-	-
	Screen high-risk populations and treat sof/velp	631	5.42	66	2.01	33
	Screen general population and treat with sof/velp	767	4.38	136	−1.04	Dominated
**USD 550**	No screening, no treatment	565	3.41	-	-	
	Screen high-risk populations and treat with sof/velp	913	5.42	348	2.01	173
	Screen general population and treat with sof/velp	1048	4.38	135	−1.04	Dominated
	**Scenario 2 sof/led**					
	No screening, no treatment	565	3.41	-	-	-
	Screen high-risk populations and treat with sof/led	1504	5.36	939	1.95	481
	Screen general population and treat with sof/led	1629	4.34	125	−1.02	dominated

ICER—Incremental cost effectiveness ratio, USD/QALY gained, QALY—Quality-adjusted year, sof/velp—sofosbuvir/velpatasvir, sof/led—sofosbuvir/ledipasvir, USD—United States dollars.

## Data Availability

Data presented in this study were derived the resources available in public domain.

## Supplementary Materials

The following supporting information can be downloaded at: https://www.mdpi.com/article/10.3390/ijerph22040509/s1, Figure S1: Decision tree showing the screening of Hepatitis C; Table S1: The parameters for the base case analysis; Table S2: The micro costing of the F4, DC and HCC health states. Refs. [12,25,29,33,36,37,65,66] are cited in the Supplementary Materials.

## Abbreviations

The following abbreviations were used in this manuscript:

CEAC	Cost effectiveness acceptability curve
CET	Cost effectiveness threshold
CSW	Commercial sex workers
DAAs	Directly acting antivirals
DALY	Disability-adjusted life years
DC	Decompensated cirrhosis
DSA	Deterministic sensitivity analysis
HCC	Hepatocellular carcinoma
HCV	Hepatitis C virus
ICER	Incremental cost effectiveness ratio
LMICs	Low- to middle-income countries
MSM	Men who have sex with men
NAT	Nucleic acid test
PSA	Probabilistic sensitivity analysis
PWID	People who inject drugs
QALY	Quality-adjusted life year
RDT	Rapid diagnostic test
Sof/velp	Sofosbuvir/velpatasvir
SVR	Sustained virologic response
WHO	World Health Organization
