# Supplementary material for: Cost-Effectiveness of Screening and Treating Chronic Hepatitis C Virus Infection in Zimbabwe
Source: Int J Environ Res Public Health. 2025 Mar 27;22(4):509. doi: 10.3390/ijerph22040509 (PMC12026964; doi:10.3390/ijerph22040509)
Supplement: Supplementary file 1 [file ijerph-22-00509-s001.zip › ijerph-3435490-supplementary.pdf]

Supplementary file

Title: Cost-effectiveness of screening and treating chronic HCV in Zimbabwe.

Blessing Dzingirai[1,2], Leolin Katsidzira[3], Maarten Postma[1], Marinus van Hulst[1,4], Nyashadzaishe Mafirakureva[5]

1. Department of Health Sciences, University of Groningen, University Medical Center Groningen, Groningen, The Netherlands.
2. Department of Pharmacy and Pharmaceutical Sciences, University of Zimbabwe, Harare, Zimbabwe
3. Department of Medicine, College of Health Sciences University of Zimbabwe, Harare Zimbabwe
4. Department of Clinical Pharmacy and Toxicology, Martini Hospital, Groningen, The Netherlands.
5. Health Economics and Decision Science, School of Health and Related Research, University of Sheffield, United Kingdom.

Corresponding author: B. Dzingirai**,** Department of Health Sciences, University of Groningen, University Medical Center Groningen, Groningen, The Netherlands. Email: b.dzingirai@umcg.nl , +263772128026, orchid : //orcid.org/0000-0001-8208-7821

1.0 Decision tree

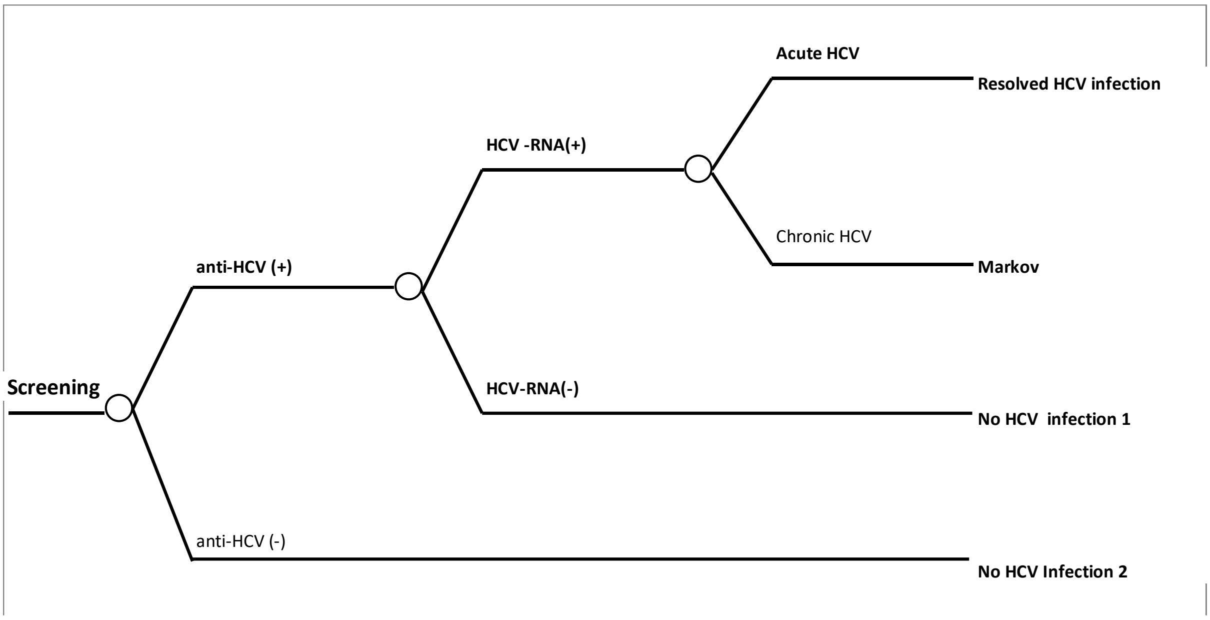

Supplementary figure S1: Decision tree showing the screening of Hepatitis C. HCV- hepatitis C virus, RNA- ribonucleic acid.

2.0 Parameters

Table S1: The Parameters for the base case analysis

Parameter Description	Deterministic	Upper limit	SE	alpha	beta	Distribution	Source
mean age patients enter the model	61	68.9					
seroprevalence of HCV in the general population	0.02	5.5%	0.01	1.57	86.57	beta	[1]
Viremic prevalence of HCV in the general population	78%	0.80	0.01	2112.10	595.72	beta	[1]
seroprevalence of HCV in the high risk populations population	4%	0.10	0.02	6.34	152.28	beta	[2]
viremic prevalence of HCV in the high risk population	78%	0.80	0.01	2112.10	595.72	beta	[2]
pooled sensitivity of the HCV RDT tests	0.98	1	0.01	736.91	15.04	beta	[3]
sensitivity of the HCV-RNA test	1	1	0.00				
proportion of individuals who spontaneously clear HCV infection	0.25	0.26	0.00	7202.75	21608.25		
Prevalence of genotype 1	0.72	0.73	0.01	2688.40	1045.49	beta	expert opinion
Prevalence of genotype 2	0.12	0.12	0.00	8623.00	63235.33	beta	expert opinion
Prevalence of genotype 3	0	0.00	0.00	0.00	0.00	beta	expert opinion

Prevalence of genotype 4	0.04	0.04	0.00	9219.80	221275.20	beta	expert opinion
Prevalence of genotype 5	0.08	0.08	0.00	8835.60	101609.40	beta	expert opinion
Prevalence of genotype 6	0.04	0.04	0.00	9219.80	221275.20	beta	expert opinion
Proportion of patients in F0	0.1	0.102	0.00	8643.50	77791.50	beta	expert opinion
Proportion of patients in F1	0.13	0.1326	0.00	8355.35	55916.57	beta	expert opinion
Proportion of patient in F2	0.15	0.153	0.00	8163.25	46258.42	beta	expert opinion
Proportion of patients in F3	0.22	0.2244	0.00	7490.90	26558.65	beta	expert opinion
Proportion of patients in F4	0.4	0.408	0.00	5762.00	8643.00	beta	expert opinion
Proportion of patients in DCC	0	0	0.00				expert opinion
Proportion of of patients in HCC	0	0	0.00				expert opinion
Proportion of patients in F0	0.89	0.9078	0.01	1055.55	130.46	beta	[4]
Proportion of patients in F1	0.08	0.0816	0.00	8835.60	101609.40	beta	[4]
Proportion of patient in F2	0.02	0.0204	0.00	9411.90	461183.10	beta	[4]
Proportion of patients in F3	0.008	0.00816	0.00	9527.16	1181367.84	beta	[4]
Proportion of patients in F4	0.002	0.00204	0.00	9584.79	4782810.21	beta	[4]
Proportion of patients in DCC	0	0	0.00				expert opinion
Proportion of of patients in HCC	0	0	0.00				expert opinion
Annual transition probability for patient from F0 to F1	0.101	0.111	0.00	390.26	3473.71	beta	[5]
Annual transition probability for	0.079	0.087	0.00	344.94	4021.42	beta	[5]

patient from F1 to F2							
Annual transition probability for patient from F2 to F3	0.11	0.121	0.01	413.59	3346.33	beta	[5]
Annual transition probability for patient from F3 to F4	0.109	0.123	0.01	282.30	2307.62	beta	[5]
Annual transition probability for patient from F4 to DCC	0.041	0.05	0.00	96.72	2262.40	beta	[6]
Annual transition probability for patient from F4 to HCC	0.042	0.051	0.00	89.81	2048.57	beta	[6]
Annual transition probability for patient from DCC to HCC	0.068	0.091	0.01	37.47	513.60	beta	[6]
Annual transition probability for patient from DCC to Death	0.13	0.163	0.01	71.91	481.28	beta	[6]
Annual transition probability for patient from HCC to death	0.9	0.94	0.02	193.58	21.51	beta	[6]
Annual utility scores for patients in state F0-F3	0.74	0.767	0.01	910.47	319.89	beta	[6]
Annual utility scores for patients in state F4	0.71	0.732	0.17	4.40	1.80	beta	[6]
Annual utility scores for patients in state DCC	0.66	0.684	0.01	1174.87	605.24	beta	[6]
Annual utility scores for patients in state HCC	0.66	0.684	0.01	1174.87	605.24	beta	[6]

Annual utility scores for patients in state F0-F3	0.78	0.807	0.01	1061.60	299.42	beta	
Annual utility scores for patients in state F4	0.75	0.771	0.01	1224.25	408.08	beta	
Annual utility scores for patients in state F0-F3	0.81	0.826	0.01	1085.11	254.53	beta	[6]
Annual utility scores for patients in state F4	0.81	0.826	0.01	1085.11	254.53	beta	[6]
Annual utility scores for patients in state DCC	0.66	0.684	0.01	1174.87	605.24	beta	[6]
Annual utility scores for patients in state HCC	0.66	0.684	0.01	1174.87	605.24	beta	[6]
Cost of treatment per individual for the treatment in F0 state	219	223.38	2.23	9604	0.02	gamma	[7]
Cost of treatment per individual for the treatment in F1 state	219	223.38	2.23	9604	0.02	gamma	[7]
Cost of treatment per individual for the treatment in F2 state	219	223.38	2.23	9604	0.02	gamma	[7]
Cost of treatment per individual for the treatment in F3 states	219	223.38	2.23	9604	0.02	gamma	[7]
Cost of treatment per individual for the treatment in F4 state	219	223.38	2.23	9604	0.02	gamma	[7]
Cost of treatment per individual for the treatment of DCC state	0	0	0.00			gamma	

Cost of treatment per individual for the treatment of HCC state	0	0	0.00			gamma	
Cost of sofosbuvir/velpatasvir 12w course per patient	1410	1438.2	14.39	9604	0.15	gamma	[7]
Cost of sofosbuvir/ledspasvir 12w course per patient	1125	1147.5	11.48	9604	0.12	gamma	[7]
Costs of management per individual in F0-F3 states	0					gamma	expert/ microcosting
Cost of treatment per individual in F4 state	0					gamma	expert/ microcosting
Cost of treatment per individual in DCC state	1286	1311.72	13.12	9604	0.13	gamma	expert/ microcosting
Cost of treatment per individual in HCC state	1024	1044.48	10.45	9604	0.11	gamma	expert/ microcosting
Cost of drugs in management of individual in F0-f3 state -no DAA Tx	0					gamma	Expert/microcosting
Cost of drugs in management of individual in F4 state -no DAA Tx	0					gamma	Expert/microcosting
Cost of drugs in management of individual in DCC state -no DAA Tx	120	122.4	1.22	9604	0.01	gamma	Expert/microcosting
Cost of drugs in management of individual in HCC state -no DAA Tx	276	281.52	2.82	9604	0.03	gamma	Expert/micro costing

Sustained virologic response in health state F0	0.94	97.7	1.79				8
Sustained virologic response in health state F1	0.94	97.7	1.79				8
Sustained virologic response in health state F2	0.94	97.7	1.79				8
Sustained virologic response in health state F3	0.94	97.7	1.79				8
Sustained virologic response in health state F4	0.94	97.7	1.79				8
Sustained virologic response in health state DCC	0						8
Sustained virologic response in health state HCC	0						
Sustained virologic response in health state F0	0.87	91	2.04				
Sustained virologic response in health state F1	0.87	91	2.04				
Sustained virologic response in health state F2	0.87	91	2.04				
Sustained virologic response in health state F3	0.87	91	2.04				
Sustained virologic response in health state F4	0.87	91	2.04				
Sustained virologic response in health state DCC	0						
Sustained virologic response in health state HCC	0						

The discount rate for costs (in %)	0.03						
The discount rate for health effects (in %)	0.03						

3.0: The transition probabilities

The meta analysis used as the source for transition probabilities estimated fibrosis progression rates using Markov maximum likelihood estimation. Rates describe number of occurrences of an event for a given time and range from zero to infinity. Probabilities describe the like hood of an event in an individual patient over a period of time thus are different from rates and range from zero to 1. There was therefore need to convert the rates reported in the meta analysis as given by the formula below:

$$p = 1 - e^{-rt}$$

Where p is the probability of transition happening in the time t, which was a one year in the model and given the rate of progression to be r.

4.0 The micro costing of the F4, DC and HCC health states

Table S2: The micro costing of the F4, DC and HCC health states

		Number	Unit Cost/US$	Total Cost/US$
F4 (Cirrhosis)				
	**Investigations**			
	screening endoscopy	2	250	500
	**Management**			
		Total cost		***500***
**Decompensated Cirrhosis**				
	**Complications**			
	Ascites			
	jaundice			
	variceal haemorrhage			
	hepatic encelopathy			
	**Investigations**			
	LFTs	2	40	80
	Uand E	2	18	36
	HIV	1	17	17

	Hep B	1	17	17
	Cell count at differential	2	20	40
	endoscopy	1	250	250
	FBC	2	18	36
	**Management**			
	Variceal erradication	3	250	750
	Propranolol 40mg bd	12	4	48
	Spironolactone 400mg bd	12	6	72
	hospitalisation	4	15	60
	Total			***1406***
**Hepatocellular carcinoma**				
	**Investigations**			
	ultra feto protein	1	18	18
	FBC	2	18	36
	Und E	2	18	36
	CT scan	2	350	700
	LFTs	2	40	80
	HB test	1	17	17
	HIV test	1	17	17
	Total			904
	**Management**			
	Morphine	12	15	180
	Tramadol	12	8	96
	palliative care	12	10	120
				396
	Total			***1300***

6.0 References

1. Mabaya S, Munongo E, Mapako T, Marowa L, Gasasira AN, Pasipanodya JG, et al. Prevalence and trends of hepatitis B and C virus biomarkers in Zimbabwe: comparative analyses of a nation's blood-donor surveillance data and meta-analyses of population studies. Infect Dis (Lond). 2024 May 14;1–17.

2. Scheibe A, Young K, Versfeld A, Spearman CW, Sonderup MW, Prabdial-Sing N, et al. Hepatitis B, hepatitis C and HIV prevalence and related sexual and substance use risk practices among key populations who access HIV prevention, treatment and related services in South Africa: findings from a seven-city cross-sectional survey (2017). BMC Infect Dis. 2020 Dec;20(1):655.

3. Tang W, Chen W, Amini A, Boeras D, Falconer J, Kelly H, et al. Diagnostic accuracy of tests to detect Hepatitis C antibody: a meta-analysis and review of the literature. BMC Infect Dis. 2017 Nov;17(S1):695.

4. Sonderup MW, Gogela N, Nordien R, Smuts H, Korsman S, Hardie D, et al. Direct-acting antiviral therapy for hepatitis C: The initial experience of the University of Cape Town/Groote Schuur Hospital Liver Clinic, South Africa. S Afr Med J. 2020 Jan 29;110(2):112.

5. Erman A, Krahn MD, Hansen T, Wong J, Bielecki JM, Feld JJ, et al. Estimation of fibrosis progression rates for chronic hepatitis C: a systematic review and meta-analysis update. BMJ Open. 2019 Nov;9(11):e027491.

6. Boyer S, Baudoin M, Nishimwe ML, Santos M, Lemoine M, Maradan G, et al. Cost-utility analysis of four WHO-recommended sofosbuvir-based regimens for the treatment of chronic hepatitis C in sub-Saharan Africa. BMC Health Serv Res. 2022 Dec;22(1):303.

7. Dzingirai B, Katsidzira L, Mwanesani V, Postma MJ, van Hulst M, Mafirakureva N. A cost analysis of a simplified model for HCV screening and treatment at a tertiary hospital in Zimbabwe. Expert Review of Pharmacoeconomics & Outcomes Research. 2024;0(0):1–9.

8. Ren XD, Fu X, He YQ, Li CY, Guo M, Qiao M. Safety and efficacy of sofosbuvir-velpatasvir: A meta-analysis. Medicine. 2022 Oct 21;101(42):e31183.
